# Assessment of Academic Motivation and Its Impact on Self-Esteem Among Saudi Arabian Undergraduate Dental Students: A Cross-Sectional Study

**DOI:** 10.7759/cureus.77262

**Published:** 2025-01-10

**Authors:** Sanjeev B Khanagar, Abdul Salam T.A, Firas Mansour A Almoallem, Saleh Naser S Alwatban, Ahmed Mohammed S Almutairi, Naser Aref N Altamimi

**Affiliations:** 1 King Abdullah International Medical Research Center, King Saud Bin Abdulaziz University for Health Sciences, Ministry of National Guard Health Affairs, Riyadh, SAU; 2 Preventive Dental Science Department, College of Dentistry, King Saud Bin Abdulaziz University for Health Sciences, Riyadh, SAU; 3 Public Health Dentistry Department, PMS College of Dental Science and Research, Thiruvananthapuram, IND; 4 College of Dentistry, King Saud Bin Abdulaziz University for Health Sciences, Riyadh, SAU

**Keywords:** academic motivation, belief, dental students, motivation, perception, self-esteem, undergraduates

## Abstract

Background: The academic performance of students is influenced by several factors, such as academic motivation, self-directed learning, their mindset, and view of themselves.

Aim: This study aimed to assess the impact of academic motivation (AM) on self-esteem (SE) among Saudi Arabian undergraduate dental students.

Methodology: A descriptive cross-sectional study was conducted among undergraduate dental students in Riyadh, Saudi Arabia. Data were collected through online survey form using pre-validated questionnaires, which included the Academic Motivation Scale (AMS) and Rosenberg Self-Esteem Scale (RSES).

Result: A total of 240 study participants were approached to participate in this research. Out of them, 161 undergraduate dental students agreed to participate, with a response rate of 67.08%, and out of those, 78.90% reported moderate levels of AM, and 52.20% exhibited low SE. AM was significantly associated with the academic year (p = 0.001) and parental education level (p = 0.006); however, no significant relationship was observed between AM and SE (p = 0.294). Additionally, demographic and personality traits showed varying levels of influence on AM and SE, though not all were statistically significant.

Conclusion: This study concludes that, while AM is significantly influenced by factors, such as academic year and parental education level, it does not have a statistically significant relationship with SE among undergraduate dental students. These findings suggest that other cultural, psychological, or contextual variables may play a more critical role in shaping SE within this population. Interventions aimed at enhancing AM and SE should consider these broader influences to effectively support students' academic success and well-being.

## Introduction

A student's desire, as demonstrated by their approach, perseverance, and level of interest, is known as academic motivation (AM) [1]. In contrast, self-esteem (SE) is characterized as a particular mindset and view of oneself [2]. Because dental education programs are known to be demanding and students encounter a variety of difficulties, such as academic pressure, heavy workloads, insomnia, lack of time, and occasionally financial difficulties, the relationship between AM and SE in the dental education system is crucial [3]. However, dental students can overcome the majority of these obstacles if they receive enough external motivation from their concerned faculty members. This will increase their internal motivation, which will improve their academic performance and possibly raise their SE [4]. Academic achievement, which includes choosing an academic career, self-directed learning, and grade point average (GPA) level, is significantly influenced by AM [5-7]. On the other hand, low AM leads to a number of psychological problems, including stress, anxiety, depression, and burnout [8-12]. Additionally, a number of studies found that these psychological problems negatively impacted the SE of undergraduate dental students in their final year [13-17]. Additionally, the majority of dental students who had low SE did poorly academically and ultimately achieved low academic success [10]. Previous studies in the literature have explored the role of AM and SE. However, no study has investigated the relationship between these two. The findings of this study will be helpful in developing tailored Interventions for the students that can be aimed at enhancing AM and SE, which can in turn influence their academic success and well-being. Hence, the aim of this study was to assess AM and its impact on SE among Saudi Arabian undergraduate dental students using the Academic Motivation Scale of Vallerand (AMSV) and Rosenberg Self-esteem Questionnaire (RSQ), respectively. The objectives were to analyze the impact of the participant's gender, cumulative GPA, parental education level, and personality trait on their responses.

## Materials and methods

Study design

A descriptive cross-sectional study was conducted from March 01, 2024, until September 01, 2024. Before initiating the study, an ethical clearance was obtained from King Abdullah International Medical Research Center (KAIMRC) (reference number: IRB/0453/23).

Eligibility criteria

The study participants comprised first- to final-year undergraduate dental students from the College of Dentistry, King Saud Bin Abdulaziz University for Health and Science, Riyadh, Saudi Arabia.

Dental students willing to participate in the study by providing written informed consent were included in the study. Dental students who are suffering from any mental health issues or are under medication for medication for psychological issues were excluded from the study.

Sample size and sampling frame

The sample size for this study was calculated based on the reported proportion of 24.3% of SE among Saudi Arabian undergraduate dental students [3]. Based on the findings, with an expected proportion from the pilot study, a relative precision of 5%, and a confidence level of 95%, the calculated sample size was 226 participants [18].

Sampling technique

A non-probability convenience sampling technique was adopted to enroll the study participants for this study.

Data collection

Data required for this study were gathered using a self-administered standardized questionnaire. The first section of the questionnaire comprised demographic details, which included the gender, current academic level, cumulative grade point, parental education, income, and their agreement with statements related to personality traits (agreeableness/conscientiousness/extraversion/neuroticism/openness). The second section comprised the questions related to the Academic Motivation Scale, which was assessed using a prevalidated questionnaire, comprising 28 questions with a 7-point Likert scale.

The scores were calculated as follows: score 1 = does not correspond at all, score 2 = does not correspond at all - correspond a little, score 3 = correspond a little, score 4 = correspond moderately, score 5 = correspond moderately - correspond a lot, score 6 = correspond a lot, score 7 = correspond exactly.

The total of the person scores were interpreted as a score below 84 for low, 84-140 for moderate, and more than 140 for high [19].

The psychometric properties of this scale were reported in the previous studies, where Cronbach’s alpha coefficient for this scale was 0.81, which indicated a satisfactory level of internal consistency [20].

The third section comprised the questions related to the SE level, which was assessed using a prevalidated questionnaire tool comprising 10 questions with a 4-point Likert scale.

The scores were calculated as follows: for items 1, 2, 4, 6, and 7: strongly agree = 3, agree = 2, disagree = 1, strongly disagree = 0; for items 3, 5, 8, 9, and 10 (which are reversed in valence): strongly agree = 0, agree = 1, disagree = 2, strongly disagree = 3.

The total scale ranged from 0 to 30. Scores more than 25 were considered high, scores between 15 and 25 were within the moderate range; and scores below 15 suggest low SE [21].

The psychometric properties of this scale were reported in the previous studies, where Cronbach’s alpha coefficient for this scale was 0.91, which indicated a good internal consistency [22].

Study participants: The hard copies of the questionnaire were provided to the dental students willing to participate in the study and provide written informed consent. The data collection process was scheduled over a period of three months.

Statistical analysis

The data were entered in a Microsoft Excel spreadsheet, and after further data cleaning, they were transferred to SPSS software (version 29; IBM Corporation, Armonk, NY) for analysis. Descriptive statistics were calculated, and chi-square analysis was performed to measure the association of demographic details on their responses. Univariate and hierarchical linear regression analyses were performed to assess the effect of the participants’ characteristics. Statistical significance was set at p < 0.05.

## Results

In the present study, 240 dental students were approached to participate in this survey, of which 161 agreed to participate, which yielded a response rate of 67.08%.

Demographic details of the study participants

The demographic details of the study participants revealed that 67 (41.60%) were female and 94 (58.40%) were male, 40 (24.80%) were D1, 32 (19.90) were D2, 39 (24.20%) were D3, and 50 (31.10%) were D4. Eighteen (11.20%) of them have a cumulative GPA of A+, 47 (29.20%) had A, 44 (27.30%) had B+, 30 (18.60%) had B, 12 (7.50%) had C+, eight (5.00%) had C, and two (1.20%) had D+. Additionally, 33 (20.50%) of the participants' parental education level was postgraduate level, 15 (9.30) were diplomas, and 14 (8.70%) had high school degrees. A total of 65 (40.40%) of their parents’ yearly income is less than 50,000 Saudi Arabian Riyals, and 96 (59.60%) have more than 50,000 Saudi Arabian Riyals (Table 1).

**Table 1:** Demographic details of the study participants D1 = First year, D2 = Second year, D3 = Third year, D4 = Fourth year A+ = 95 to 100, A= 90 to < 95, B+ = 85 to < 90, B = 80 to < 85, C+ = 75 to < 80, C = 70 to < 75, D+ = 65 to < 70

Statements	Responses	Number	Percentage
Gender	Female	67	41.60%
	Male	94	58.40%
Current Academic Level	D1	40	24.80%
	D2	32	19.90%
	D3	39	24.20%
	D3	50	31.10%
Last Year’s Cumulative Grade Point Average (CGP)	A	47	29.20%
	A+	18	11.20%
	B	30	18.60%
	B+	44	26.30%
	C	8	5.00%
	C+	12	7.50%
	D+	2	1.20%
Parental Education Level	Graduate	99	61.50%
	High school	14	8.70%
	Intermediate/Diploma	15	9.30%
	Postgraduate	33	20.50%
Income	Less than 50,000	65	40.40%
	More than 50,000	96	59.60%

Details of AM and SE scores

In the present study, the AM scores were low for five (3.10%) study participants, moderate for 127 (78.90), and high for 29 (18.00%). The SE scores were low for 84 (52.20%) study participants, moderate for 40 (24.80%), and high for 37 (23.00%) (Table 2).

**Table 2:** Details of academic motivation scores and self-esteem scores

	Level	Number	Percentage
Academic Motivation Scores	Low academic motivation (scores < 84)	5	3.10%
	Moderate academic motivation (scores 84-140)	127	78.90%
	High academic motivation (scores > 140)	29	18.00%
Self-Esteem Scores	Low self esteem (scores < 15)	84	52.20%
	Moderate self esteem (scores 15-25)	40	24.80%
	High self esteem (scores > 25)	37	23.00%

Association between demographic details and AM scores

In the present study, the association between demographic details and AM scores was determined using chi-square analysis, which revealed that D1 students exhibited higher scores in comparison with other counterparts, and this difference was found to be statistically significant (p value = 0.01). Students whose parental education was graduate level exhibited higher scores in comparison with other counterparts, and this difference was found to be statistically significant (p value = 0.01) (Table 3).

**Table 3:** Association between demographic details and academic motivation scores * = statistically significant D1 = First year, D2 = Second year, D3 = Third year, D4 = Fourth year A+ = 95 to 100, A = 90 to < 95, B+ = 85 to < 90, B = 80 to < 85, C+ = 75 to < 80, C = 70 to < 75, D+ = 65 to < 7

		Academic Motivation Scores								
		Low academic motivation (scores < 84)		Moderate academic motivation (scores 84-140)		High academic motivation (scores > 140)			X^2^ value	p value
		N	%	N	%	N		%		
Gender	Female	0	0.00%	57	44.90%		10	34.50%	4.729	0.094
	Male	5	100.00%	70	55.10%		19	65.50%		
Last Year's Cumulative Grade Point Average (CGP)	A	2	40.00%	36	28.30%		9	31.00%	9.539	0.656
	A+	0	0.00%	15	11.80%		3	10.30%		
	B	1	20.00%	20	15.70%		9	31.00%		
	B+	2	40.00%	38	29.90%		4	13.80%		
	C	0	0.00%	6	4.70%		2	6.90%		
	C+	0	0.00%	11	8.70%		1	3.40%		
	D+	0	0.00%	1	0.80%		1	3.40%		
Current Academic Level	D1	0	0.00%	27	21.30%		13	44.80%	31.79	0.001*
	D2	5	100.00%	27	21.30%		0	0.00%		
	D3	0	0.00%	33	26.00%		6	20.70%		
	D4	0	0.00%	40	31.50%		10	34.50%		
Parent Education Level	Graduate	2	40.00%	82	64.60%		15	51.70%	18.13	0.006*
	High school	0	0.00%	10	7.90%		4	13.80%		
	Intermediate/Diploma	3	60.00%	9	7.10%		3	10.30%		
	Postgraduate	0	0.00%	26	20.50%		7	24.10%		
Personality Trait	Agreeableness ( Tendency to agree and go with others rather than assert own opinion)	0	0.00%	15	11.80%		7	24.10%	11.286	0.186
	Conscientiousness (Tendency to be careful, on time for appointments, follow rules, hardworking)	3	60.00%	45	35.40%		7	24.10%		
	Extraversion (Tendency to be talkative, sociable, and to enjoy others)	1	20.00%	31	24.40%		12	41.40%		
	Neuroticism (Tendency to frequently experience negative emotions such as anger and sadness)	0	0.00%	7	5.50%		1	3.40%		
	Openness (Tendency to appreciate new art, ideas, values feelings, and behaviors)	1	20.00%	29	22.80%		2	6.90%		

Association between demographic details and SE scores

In the present study, the association between demographic details and SE scores was performed using chi-square analysis, which revealed that D3 students exhibited higher scores in comparison with other counterparts, and this difference was found to be statistically significant (p value = 0.01) (Table 4).

**Table 4:** Association between demographic details and self-esteem scores * = statistically significant D1 = First year, D2 = Second year, D3 = Third year, D4 = Fourth year A+ = 95 to 100, A = 90 to < 95, B+ = 85 to < 90, B = 80 to < 85, C+ = 75 to < 80, C = 70 to < 75, D+ = 65 to < 7

		Self-Esteem Score								
		Low self-esteem (scores < 15)		Moderate self-esteem (scores 15-25)		High self-esteem (scores > 25)			X^2 ^ value	p value
		N	%	N	%	N		%		
Gender	Female	38	45.20%	15	37.50%		14	37.80%	0.95	0.622
	Male	46	54.80%	25	62.50%		23	62.20%		
Last Year's Cumulative Grade Point Average (CGP)	A	30	35.70%	10	25.00%		7	18.90%	15.162	0.233
	A+	10	11.90%	5	12.50%		3	8.10%		
	B	11	13.10%	12	30.00%		7	18.90%		
	B+	20	23.80%	9	22.50%		15	40.50%		
	C	4	4.80%	3	7.50%		1	2.70%		
	C+	7	8.30%	1	2.50%		4	10.80%		
	D+	2	2.40%	0	0.00%		0	0.00%		
Current Academic Level	D1	33	39.30%	7	17.50%		0	0.00%	69.862	0.001*
	D2	25	29.80%	5	12.50%		2	5.40%		
	D3	2	2.40%	13	32.50%		24	64.90%		
	D4	24	28.60%	15	37.50%		11	29.70%		
Parental Education Level	Graduate	54	64.30%	20	50.00%		25	67.60%	3.674	0.721
	High school	6	7.10%	5	12.50%		3	8.10%		
	Intermediate/Diploma	8	9.50%	5	12.50%		2	5.40%		
	Post Graduate	16	19.00%	10	25.00%		7	18.90%		
Personality Trait	Agreeableness (Tendency to agree and go with others rather than assert own opinion)	16	19.00%	3	7.50%		3	8.10%	10.846	0.211
	Conscientiousness (Tendency to be careful, on time for appointments, to follow rules, hardworking)	26	31.00%	18	45.00%		11	29.70%		
	Extraversion (Tendency to be talkative, sociable, and to enjoy others)	22	26.20%	10	25.00%		12	32.40%		
	Neuroticism (Tendency to frequently experience negative emotions such as anger and sadness)	3	3.60%	4	10.00%		1	2.70%		
	Openness (Tendency to appreciate new art, ideas, values feelings, and behaviors)	17	20.20%	5	12.50%		10	27.00%		

Association between SE and AM scores

In the present study, the association between SE scores and AM scores was determined using chi-square analysis, which revealed that there was no association between the two (Table 5).

**Table 5:** Association between self-esteem scores and academic motivation scores

		Academic Motivation Scores								
		Low academic motivation (scores < 84)			Moderate academic motivation (scores 84-140)		High academic motivation (scores > 140		X^2^ value	p value
		N		%	N	%	N	%		
Self-Esteem Score (SES)	Low SES (scores < 15)	5	100.00%		64	50.40%	15	51.70%	4.935	0.294
	Moderate SES (scores 15-25)	0	0.00%		32	25.20%	8	27.60%		
	High score SES (scores > 25)	0	0.00%		31	24.40%	6	20.70%		

The effect of characteristics intermediate variables

In the present study, univariate and hierarchical linear regression analyses were performed. The results of the regression analysis are presented in seven blocks, which did not show any statistically significant positive or negative effect on participants' characteristics in the study (Table 6).

**Table 6:** The effect of characteristics intermediate variables *Statistical significance set at p<0.05; Predictors: (Constant), Self-Esteem; Dependent Variable: Academic Motivation

Block	Level and variable type	Regression coefficient (standard error)	P value	Results of Regression
1	Self-Esteem (Score 3)			R^2^=0.01, Adjusted R^2^=0.01
	Score 2	0.26 (0.6)	0.668	
	Score 1	0.12 (0.53)	0.826	
2	Self-Esteem (Score 3)			R^2^=0.01, Adjusted R^2^=0.02
	Score 2	0.22 (0.6)	0.715	
	Score 1	0.0 (0.54)	0.995	
3	Self-Esteem (Score 3)			R^2^=0.02, Adjusted R^2^=0.03
	Score 2	0.21 (0.6)	0.723	
	Score 1	0.02 (0.54)	0.967	
	Gender (Male)	-0.34 (0.43)	0.434	
4	Self-Esteem (Score 3)			R^2^=0.07, Adjusted R^2^=0.12
	Score 2	0.04 (0.63)	0.943	
	Score 1	-0.2 (0.58)	0.736	
	Gender (Male)	-0.28 (0.47)	0.551	
	Last year’s cumulative grade (A)			
	CGP A+	-0.37 (0.76)	0.626	
	CGP B	0.74 (0.58)	0.203	
	CGP B+	-0.84 (0.66)	0.202	
	CGP C	0.101 (0.98)	0.299	
	CGP C+	0.26 (1.18)	0.823	
	CGP D+	2.3 (1.59)	0.147	
5	Self-Esteem (Score 3)			R^2^=0.16, Adjusted R^2^=0.26
	Score 2	-0.27 (0.7)	0.698	
	Score 1	-0.54 (0.81)	0.507	
	Gender (Male)	-0.26 (0.5)	0.597	
	Last year’s cumulative grade (A)			
	CGP A+	-0.109 (0.79)	0.167	
	CGP B	0.37 (0.64)	0.564	
	CGP B+	-0.108 (0.69)	0.117	
	CGP C	0.29 (1.03)	0.781	
	CGP C+	-0.101 (1.23)	0.412	
	CGP D+	1.31 (1.62)	0.42	
	Current level (D1)			
	D2	-21.73 (7251.17)	0.998	
	D3	-1.55 (1.12)	0.168	
	D4	-1.03 (1.45)	0.478	
6	Self-Esteem (Score 3)			R^2^=0.17, Adjusted R^2^=0.27
	Score 2	-0.33 (0.72)	0.642	
	Score 1	-0.48 (0.83)	0.56	
	Gender (Male)	-0.24 (0.5)	0.626	
	Last year’s cumulative grade (A)			
	CGP A+	-1.03 (0.8)	0.198	
	CGP B	0.35 (0.64)	0.586	
	CGP B+	-1.12 (0.69)	0.108	
	CGP C	0.44 (1.05)	0.674	
	CGP C+	-1.19 (1.3)	0.362	
	CGP D+	0.97 (1.73)	0.577	
	Current level (D1)			
	D2	-22.12 (7187.85)	0.998	
	D3	1.49 (1.14)	0.189	
	D4	-1.31 (1.43)	0.366	
	Parent’s education (Postgraduate)			
	High school	0.51 (0.79)	0.524	
	Intermediate/Diploma	1.31 (1.01)	0.197	
	Graduate	0.05 (0.58)	0.935	
7	Self-Esteem (Score 3)			R^2^=0.17, Adjusted R^2^=0.27
	Score 2	-0.43 (0.73)	0.556	
	Score 1	-0.59 (0.83)	0.477	
	Gender (Male)	-0.17 (0.5)	0.734	
	Last year’s cumulative grade (A)			
	CGP A+	-1.15 (0.81)	0.157	
	CGP B	0.39 (0.64)	0.545	
	CGP B+	-1.21 (0.7)	0.087	
	CGP C	0.39 (1.07)	0.717	
	CGP C+	-1.31 (1.31)	0.319	
	CGP D+	1.03 (1.69)	0.544	
	Current level (D1)			
	D2	-21.98 (7207.25)	0.998	
	D3	-1.7 (1.15)	0.139	
	D4	-1.35 (1.46)	0.354	
	Parent’s education (Postgraduate)			
	High school	0.94 (0.88)	0.281	
	Intermediate/Diploma	1.35 (1.04)	0.194	
	Graduate	0.31 (0.62)	0.613	

## Discussion

This study explored the relationship between AM and SE among Saudi undergraduate dental students. Our findings reveal a complex interplay between these two constructs, but contrary to initial expectations, no statistically significant association was observed between AM and SE [4]. This outcome warrants a deeper exploration of the factors influencing both AM and SE in the unique context of dental education [5].

Our analysis showed that AM levels among the participants were predominantly moderate, with significant associations observed with the academic year and parental education level. These findings align with existing literature suggesting that academic year progression often influences motivation, as senior students face increased academic demands and professional expectations [13]. For instance, D4 (final-year) students exhibited the highest levels of motivation, potentially reflecting a greater sense of purpose and proximity to career goals [10]. Parental education also emerged as a significant factor, with students from more educated families exhibiting higher levels of motivation [18]. This finding underscores the role of socioeconomic and familial support in fostering academic commitment [18]. Parents' education may influence a student’s intrinsic drive by shaping aspirations and providing a supportive learning environment. The observed correlation aligns with prior research highlighting the positive impact of parental involvement on AM across various educational settings [6].

The study found that SE levels were generally low among the participants, with significant variations based on the academic year. Interestingly, senior students’ demonstrated higher SE compared to their junior counterparts, possibly reflecting increased confidence gained through academic and clinical experiences [3]. However, the high prevalence of low SE across the cohort raises concerns, particularly given its well-documented link to mental health challenges, such as depression and anxiety, in dental students [17]. Our findings resonate with studies suggesting that the rigorous nature of dental education often places students under immense psychological strain, which may adversely affect their SE [17]. Factors such as academic pressure, competition, and the fear of failure are known contributors to low SE in dental programs [15]. These insights underscore the need for targeted interventions to support students’ psychological well-being throughout their academic journey.

Despite theoretical underpinnings suggesting a potential link between AM and SE, our study did not find a statistically significant association between the two variables. This outcome deviates from findings in other educational contexts, such as nursing and medical students, where a positive relationship has been documented [17]. Several factors may explain this discrepancy.

First, cultural influences unique to Saudi Arabia could play a pivotal role. The Saudi educational system emphasizes collective values, which may affect individual perceptions of self-worth and motivation differently compared to Western contexts. For instance, societal expectations and familial obligations may lead students to prioritize external validation over intrinsic motivation, potentially weakening the link between AM and SE [11]. Second, the nature of the dental profession itself may contribute to the observed outcome. Dental education is characterized by high levels of stress, with students often facing significant challenges, such as heavy workloads, demanding clinical responsibilities, and frequent assessments [11]. These stressors may overshadow the potential positive effects of motivation on SE, particularly among students who struggle to meet these demands [11]. Third, personality traits and psychological factors not accounted for in this study may mediate the relationship between AM and SE. For example, traits such as resilience, emotional intelligence, and self-efficacy are known to influence both motivation and SE [4]. Future research could benefit from incorporating these variables to better understand their interplay.

The findings of this study have important implications for educators and administrators in dental education. Given the significant influence of parental education on AM, institutions may consider engaging parents in their students’ academic journeys through outreach programs or workshops [6]. Additionally, the high prevalence of low SE among dental students highlights the need for comprehensive mental health support systems, including counseling services and stress management programs [15]. Moreover, our findings suggest that interventions targeting motivation and SE should be tailored to the specific needs of students at different academic levels. For example, junior students may benefit from mentorship programs that help them navigate the transition to dental school, while senior students could be supported through career counseling and professional development opportunities [10].

This study has a few limitations that should be acknowledged. The cross-sectional design limits the ability to infer causal relationships between variables, and the sample was restricted to a single institution, potentially limiting the generalizability of the findings. Additionally, the reliance on self-reported measures may introduce response bias, as participants may have provided socially desirable answers. Future research should address these limitations by employing longitudinal designs and incorporating more diverse samples from multiple institutions. Investigating additional factors, such as cultural norms, psychological traits, and coping mechanisms, could also provide a more comprehensive understanding of the relationship between AM and SE. Furthermore, exploring the long-term impact of motivation and SE on academic and professional outcomes could produce valuable insights for curriculum development and student support strategies.

## Conclusions

This study found that AM was influenced by factors such as academic year and parental education but showed no direct association with SE. The high prevalence of low SE highlights the need for mental health support and tailored interventions. Future research should explore cultural and psychological factors to better address students’ academic and emotional well-being.
